# Supplementary material for: Experiences of oral pre-exposure prophylaxis (PrEP) use disclosure among South African adolescent girls and young women and its perceived impact on adherence
Source: PLoS One. 2021 Mar 5;16(3):e0248307. doi: 10.1371/journal.pone.0248307 (PMC7935254; doi:10.1371/journal.pone.0248307)
Supplement: S1 File — (DOCX) [file pone.0248307.s001.docx]

# Supporting information

##

## S1 File. Qualitative IDI Guide (Disclosure and Relationships)

### Disclosure:

1. **Did you tell anyone in your life that you are using PrEP?**
   - *If yes:*
     - Who did you tell?
     - Why did you tell them?
     - How did they respond? *(Probe about family members, partners, friends, others)*
     - Did you experience any problems after telling them?
     - Is there anyone else you are planning to tell?
   - *If no:*
     - Why haven’t you spoken to anyone about PrEP? What concerns do you have about telling others?
     - Is there anyone you would like to share this with?
     - How do you think they would respond, if you told them?
     - Do you think you will?
   - Did you discuss whether/how to tell people in your life about PrEP with anyone here?
     - *If yes:* Any feedback about the counselling you received?
     - *If no:* What kind of support would be helpful?
   - Does anyone support you around taking PrEP? What do they do, if so?
     - Is there any kind of support you feel you need, but do not have?
2. **Have you given the brochure to anyone to read**?
   - If yes, who did you give it to? Tell me about what you discussed…
   - Did the brochure make it any easier to talk about PrEP with that person? How so?

### Boyfriend/intimate sex partner(s):

1. *(if she shared about a partner at the previous interview)* **Are you still seeing the same partner?**
   - *If yes,* how do you feel about how the relationship is going lately? Do you have any concerns about it?
     - *(be mindful of her previous answers when asking)* Do you have other partners? Do you think your partner(s) have other sexual partners? What makes you think that? How do you feel about that? Have you discussed it? *(describe, if so)*
   - *If no,* what happened? *(then move to the next question)*
2. *(if she didn’t say she had a partner last time)* **Do you have a sex partner at the moment? Tell me about your relationship** *(Probe about partner / relationship characteristics)*
   - How long have you been seeing them? How old are they? How did you meet?
   - How would you describe your relationship? How do you feel about how the relationship is going lately? Do you have any concerns about it? What is the main reason you are with this partner?
   - Do you and your partner ever use condoms? What influences whether you use them or not *(when would you use one and when wouldn’t you?)*
   - Do you have other partners? Do you think your partner(s) have other sexual partners? What makes you think that? How do you feel about that? Have you discussed it? *(describe, if so)*
3. **Have you spoken to your partner(s) about HIV? What have you discussed?**
   - How do you think he views HIV—do you think he thinks about his own risk much? What makes you say that?
   - Have you told your partner about PrEP?
     - *If yes:* What did you say? How did he respond? How does your partner feel about your taking PrEP?
     - *If no:* How do you think he/she would feel about PrEP?
   - Has being in the study influenced your relationship? Have you had any conversations that you might not have had otherwise?
     - Have you discussed testing with your partner(s)? Has he tested? Do you know his (or her) HIV status?
     - Did his agreeing (or refusing) to test affect how much you worry about HIV? Or your motivation to use PrEP? (*Does knowing / not knowing whether or not he has HIV affect your motivation to use PrEP?)*
4. **Violence**—**Have you experienced any violence in your relationship(s)?** *(Probe about forms of violence: psychological, emotional, sexual, physical)*
   - If so, did that have any effect on your PrEP use?
5. **Has using PrEP impacted your life in any other ways?** *(Probe about relationship dynamics, self-confidence, intimacy/trust, other benefits/challenges)*
   - Is there anything different about your life now, than before you joined 3P?
